# Hotspots and trends in ophthalmology in recent 5 years: Bibliometric analysis in 2017–2021

**DOI:** 10.3389/fmed.2022.988133

**Published:** 2022-08-26

**Authors:** Yuan Tan, Weining Zhu, Yingshi Zou, Bowen Zhang, Yinglin Yu, Wei Li, Guangming Jin, Zhenzhen Liu

**Affiliations:** ^1^State Key Laboratory of Ophthalmology, Zhongshan Ophthalmic Center, Sun Yat-sen University, Guangzhou, China; ^2^Guangdong Provincial Key Laboratory of Ophthalmology and Visual Science, Guangzhou, China; ^3^Guangdong Provincial Clinical Research Center for Ocular Diseases, Guangzhou, China; ^4^Zhongshan Medical School, Sun Yat-sen University, Guangzhou, China

**Keywords:** ophthalmology, hotspots, research trend, bibliometric analysis, literature

## Abstract

**Purpose:**

The purpose of this study was to investigate the hotspots and research trends of ophthalmology research.

**Method:**

Ophthalmology research literature published between 2017 and 2021 was obtained in the Web of Science Core Collection database. The bibliometric analysis and network visualization were performed with the VOSviewer and CiteSpace. Publication-related information, including publication volume, citation counts, countries, journals, keywords, subject categories, and publication time, was analyzed.

**Results:**

A total of 10,469 included ophthalmology publications had been cited a total of 7,995 times during the past 5 years. The top countries and journals for the number of publications were the United States and the Ophthalmology. The top 25 global high-impact documents had been identified using the citation ranking. Keyword co-occurrence analysis showed that the hotspots in ophthalmology research were epidemiological characteristics and treatment modalities of ocular diseases, artificial intelligence and fundus imaging technology, COVID-19-related telemedicine, and screening and prevention of ocular diseases. Keyword burst analysis revealed that “neural network,” “pharmacokinetics,” “geographic atrophy,” “implementation,” “variability,” “adverse events,” “automated detection,” and “retinal images” were the research trends of research in the field of ophthalmology through 2021. The analysis of the subject categories demonstrated the close cooperation relationships that existed between different subject categories, and collaborations with non-ophthalmology-related subject categories were increasing over time in the field of ophthalmology research.

**Conclusions:**

The hotspots in ophthalmology research were epidemiology, prevention, screening, and treatment of ocular diseases, as well as artificial intelligence and fundus imaging technology and telemedicine. Research trends in ophthalmology research were artificial intelligence, drug development, and fundus diseases. Knowledge from non-ophthalmology fields is likely to be more involved in ophthalmology research.

## Introduction

More than 2.2 billion people worldwide were visually impaired or blind to date, with an annual economic burden of more than $269.4 billion (1). Development in ophthalmology is essential for the prevention and treatment of eye diseases, and relevant research is growing rapidly in breadth and depth and forming complex knowledge networks. Glaucoma, age-related macular degeneration, and some hereditary eye diseases were previously considered irreversible blindness-causing diseases, and progress had been made to cure or alleviate them by modulating new targets or using new technologies (2–4). Cataracts and posterior capsular opacification were previously thought to be treated only with surgery, but in the recent years, there had been new developments in research into drugs that inhibit cataract formation (5, 6). With the advances in the field of ophthalmology, new hope has emerged in areas previously considered untreatable or treatable only through non-pharmaceutical interventions (7–10). However, it is not feasible to analyze the overall overview of the field of ophthalmology and to explore its research hotspots and trends with a traditional systematic review, which is not conducive to the development of the field.

Bibliometric analysis is the quantitative analysis of the universal scientific production data in a specific field (11). Bibliometric method obtains the history and current status of the research field development by analyzing the scientific research results and can make predictions of the research field (12). Previous studies have conducted bibliometric analysis on individual country contributions or focused only on randomized controlled studies in ophthalmology and citation patterns in ophthalmology journals (13–19). Unsolved questions still remain as to how to quantitatively evaluate the contribution of different global research forces (countries, journals) in ophthalmology and identify hotspots and future research trends in ophthalmology based on a wide range of research results in different subfields of ophthalmology.

This study was intended to quantitatively analyze and visualize the global ophthalmology publication from 2017 to 2021 using bibliometric methods to explore the global research forces (countries, journals), possible hotspots, and future trends of ophthalmology research and to provide insight for research development and public health policy formulation in the field of ophthalmology.

## Methods

### Data sources

All the data used in this study were obtained from the Web of Science Core Collection (Clarivate Analytics, Philadelphia, PA, USA). The search was conducted by searching the Topic Subject retrieval field using “ophthalmology” as the subject word. Articles published between 2017 and 2021 were included, with no restrictions on the language type or document type of the articles. Data were collected on 28 January 2022.

### Data collection and processing

To describe the number of articles published per year, the number of annual citations of the articles, the number of country publications, and the number of journal publications in the field of ophthalmology, relevant data were downloaded in the Web of Science Core Collection. All ophthalmology-related articles with their corresponding references and all publication-related information were exported as plain text for country collaboration analysis, keyword co-occurrence analysis, keyword burst analysis, and subject category co-occurrence analysis. To make the results more informative, keywords that were not relevant or meaningful to the analysis were filtered and removed during the data processing.

### Statistical and bibliometric analysis

Statistical descriptions of the number of annual publications, the number of annual citations, the number of country publications, and the number of journal publications were performed using Microsoft Excel 2019 (Microsoft Corporation, Redmond, WA, USA) and GraphPad Prism version 8.4.2 (GraphPad Software, La Jolla, CA, USA).

Bibliometric analysis was carried out using VOSviewer (Leiden University's Centre for Science and Technology Studies, Leiden, the Netherlands) to obtain country collaborations and research hotspots. Several clusters were formed based on the country cooperation analysis, with countries of the same color belonging to the same cluster. Countries within clusters cooperated relatively closely, whereas cooperation among countries between clusters was relatively weak. The research hotspots were obtained from the clusters formed by the co-occurrence analysis of high-frequency keywords. The common characteristics of high-frequency keywords within the same cluster revealed the research hotspots. The frequency of keyword occurrences was used to weight the size of the keywords. The larger the keyword, the higher the frequency of occurrence.

Furthermore, CiteSpace V version 5.8.R3 (Drexel University, Philadelphia, PA, USA) was used for bibliometric analysis to obtain the burst keywords and subject category cooperation. The keyword burst analysis was performed to obtain temporal trends in keywords in the field of ophthalmology. The most recent burst keywords were defined as research frontier topics, indicating the potential for continued research breakthroughs in these topics. The co-occurrence of subject categories was analyzed to obtain the collaboration of subject categories. The number of occurrences of a subject category was used to weight the subject category. The more occurrences a subject category had, the larger it was. Temporal trends in subject category occurrences were represented by temporal rings of subject categories, the thickness of which represented the number of subject category occurrences in the corresponding year. Interdisciplinary cooperation was represented by the connecting line between subject categories. The thicker the connecting line, the closer the collaboration.

## Results

### Global research output distribution

**Figure 1:**
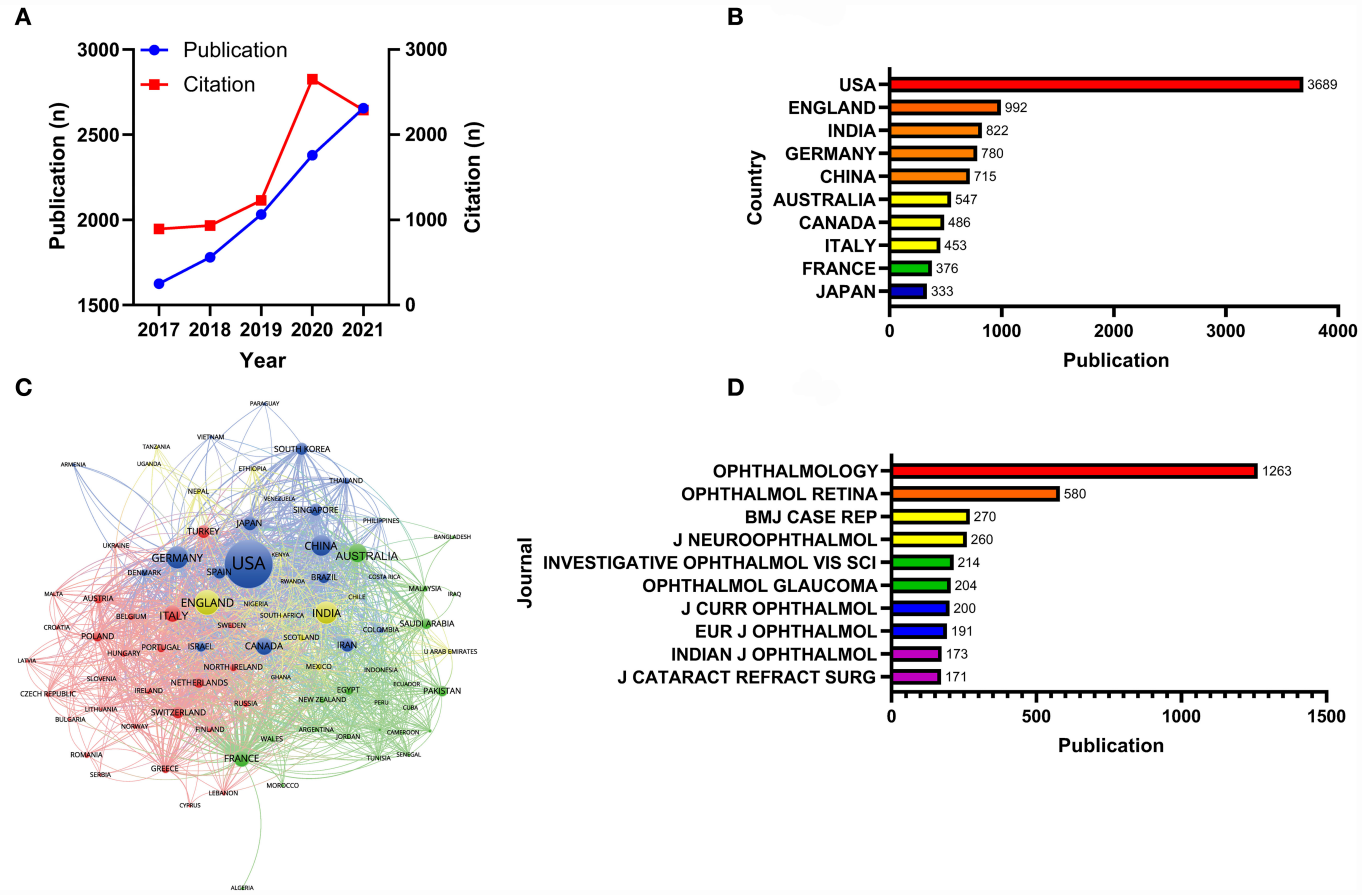
Global distribution of research output. **(A)** Annual publications and citations of ophthalmology research from 2017 to 2021. **(B)** Top 10 countries in terms of total publications. **(C)** Country cooperation networks. **(D)** Top 10 journals by total publication volume of ophthalmology research in a 5-year period.

A total of 139 countries contributed to the publications related to ophthalmology research, with a total of 10,469 articles, which were cited 7,995 times. The number of publications had increased year by year, but there was an inflection point in citation counts. Citation counts increased year by year from 2017, reaching 2,650 citations in 2020, whereas citations in 2021 decreased compared to 2020 (Figure 1A). The analysis of countries showed that the United States had the highest number of publications, more than three to four times the number of other countries, followed by the United Kingdom, India, Germany, and China (Figure 1B). Country collaboration analysis yielded four clusters, with close cooperation between countries within each cluster (Figure 1C). Publications related to ophthalmology research were distributed in 1,876 journals, and the top 10 journals in terms of the number of articles published were the Ophthalmology (*n* = 1,263, 12.06%), the Ophthalmology. Retina (*n* = 580, 5.54%), the BMJ Case Reports (*n* = 270, 2.58%), the Journal of Neuro-Ophthalmology: the official journal of the North American Neuro-Ophthalmology Society (*n* = 260, 2.48%), the Investigative Ophthalmology & Visual Science (*n* = 214, 2.04%), the Ophthalmology, Glaucoma (*n* = 204, 1.95%), the Journal of Current Ophthalmology (*n* = 200, 1.91%), the European Journal of Ophthalmology (*n* = 191, 1.82%), the Indian Journal of Ophthalmology (*n* = 173, 1.65%), and Journal of Cataract and Refractive Surgery (*n* = 171, 1.63%) (Figure 1D).

### Global high-impact documents

The top 25 high-impact articles in ophthalmology published between 2017 and 2021, ranked by total citations, are shown in Table 1. All the articles had been cited more than 150 times, with the highest number of citations being 419. Of these articles, 10 were published in 2017, 12 in 2018, one in 2019, and two in 2020. In total, 12 of these articles were published in the Ophthalmology and three in the Progress in Retinal and Eye Research. According to the type of publication, there were 16 original research articles and 9 review articles. The keywords involved in the articles are listed in Table 1, including 5 articles each on OCT and deep learning, 4 articles each on diabetes and macular degeneration, and other related research topics such as glaucoma, artificial intelligence, and drugs.

**Table 1:** Top 25 most cited documents published between 2017 and 2021.

**Author**	**Times**	**Article title**	**Document**	**Keywords**	**Journal**	**Publication**
	**cited**		**type**		**abbreviation**	**time**
Gargeya and Leng	419	Automated Identification of Diabetic Retinopathy Using Deep Learning	Article	Computer-aided diagnosis; retinal images; discrimination; system;	OPHTHALMOLOGY	2017.7
Kashani et al.	355	Optical coherence tomography angiography: A comprehensive review of current methods and clinical applications	Review	Foveal avascular zone; swept-source OCT; indocyanine green angiography; blood flow velocity; diabetic macular edema; retinal vein occlusion; amplitude decorrelation angiography; radial peripapillary capillaries; subretinal hyper-reflective material; spectral domain; optical coherence tomography angiography; retina; glaucoma; physiology; vascular disease; macular degeneration	PROG RETIN EYE RES	2017.9
Hatemi et al.	276	2018 update of the EULAR recommendations for the management of Behcet's syndrome	Review	Long-term efficacy; anti-TNF-alpha; intravitreal triamcinolone acetonide; human recombinant interferon-alpha-2a; pulmonary artery involvement; nervous system symptoms; cystoid macular edema; double-blind; refractory uveitis; extraocular manifestations; Behcet's disease; anti-TNF; treatment	ANN RHEUM DIS	2018.6
Ting et al.	256	Artificial intelligence and deep learning in ophthalmology	Review	Major risk factors; diabetic retinopathy; global prevalence; macular degeneration; automatic segmentation; intraocular pressure; glaucoma progression; neural networks; retinal layer; prematurity; imaging; retina; glaucoma; telemedicine; public health	BRIT J OPHTHALMOL	2019.2
Li et al.	237	Efficacy of a Deep Learning System for Detecting Glaucomatous Optic Neuropathy Based on Color Fundus Photographs	Article	Open-angle glaucoma; diabetic retinopathy; global prevalence; population; features; disc; impairment; strategies; diagnosis; blindness;	OPHTHALMOLOGY	2018.8
Del Amo et al.	235	Pharmacokinetic aspects of retinal drug delivery	Article	Endothelial growth factor; single intravitreal injection; cystoid macular edema; blood aqueous barrier; serum albumin nanoparticles; ocular tissue distribution; cell-penetrating peptide; inner limiting membrane; neonatal fc-receptor; human vitreous humor; retina; vitreous; choroid; topical; intravitreal; sub-conjunctival; suprachoroidal; clearance; distribution; pharmacokinetic modeling; transport	PROG RETIN EYE RES	2017.3
Kuriyan et al.	223	Vision Loss after Intravitreal Injection of Autologous Stem Cells for AMD	Article	*In vitro* differentiation; optic nerve diseases; macular degeneration; ophthalmology treatment; clinics; interventions; FDA; therapies; scots;	NEW ENGL J MED	2017.3
Sadda et al.	222	Consensus Definition for Atrophy Associated with Age-Related Macular Degeneration on OCT Classification of Atrophy Report 3	Article	Optical coherence tomography; subretinal drusenoid deposits; geographic atrophy; fundus autofluorescence; predictive value; grading system; end points; maculopathy; progression; growth;	OPHTHALMOLOGY	2018.4
Chen and Wang	216	Optical coherence tomography based angiography [Invited]	Article	Retinal vein occlusion; amplitude decorrelation angiography; macular telangiectasia type-2; swept-source OCT; flow velocity estimation; cerebral blood flow; *in vivo*; spectral domain; human skin; micro-angiography;	BIOMED OPT EXPRESS	2017.2
Lee et al.	215	Deep Learning Is Effective for Classifying Normal versus Age-Related Macular Degeneration OCT Images	Article	NA	OPHTHALMOL RETINA	2017.7
Dugel et al.	207	HAWK and HARRIER: Phase 3, Multicenter, Randomized, Double-Masked Trials of Brolucizumab for Neovascular Age-Related Macular Degeneration	Review	Visual acuity loss; treat-and-extend; intravitreal ranibizumab; aflibercept; bevacizumab; management; outcomes; therapy; safety;	OPHTHALMOLOGY	2020.1
Lai et al.	204	Stepping up infection control measures in ophthalmology during the novel coronavirus outbreak: an experience from Hong Kong	Review	Coronavirus; COVID-19; Hong Kong; infection control; ophthalmology; SARS-CoV-2;	GRAEF ARCH CLIN EXP	2020.5
Schmidt-Erfurth et al.	199	Artificial intelligence in retina	Article	Optical coherence tomography; diabetic macular edema; fully automated detection; visual field thresholds; deep learning algorithm; anti-VEGF therapy; treat-and-extend; SD-OCT; geographic atrophy; neural network; artificial intelligence (AI); machine learning (ML); deep learning (DL); automated screening; prognosis and prediction; personalized healthcare (PHC)	PROG RETIN EYE RES	2018.11
Melles et al.	191	Accuracy of Intraocular Lens Calculation Formulas	Article	Biometry; SRK/T; eyes;	OPHTHALMOLOGY	2018.2
Yamane et al.	187	Flanged Intrascleral Intraocular Lens Fixation with Double-Needle Technique	Article	Scleral fixation; anterior chamber; follow-up; open-loop; implantation; suture; eyes; complications; management; capsules;	OPHTHALMOLOGY	2017.8
Colijn et al.	180	Prevalence of Age-Related Macular Degeneration in Europe	Article	Optical coherence tomography; endothelial growth factor; beaver dam eye; visual impairment; heart disease; birth cohort; maculopathy; population; blindness; trends;	OPHTHALMOLOGY	2017.12
Moccia et al.	179	Blood vessel segmentation algorithms - Review of methods, datasets and evaluation metrics	Review	Oriented flux symmetry; active contour model; retinal images; computed tomography; lumen segmentation; minimal paths; front propagation; neural networks; fast extraction; level; blood vessels; medical imaging; review; segmentation	COMPUT METH PROG BIO	2018.5
Wu et al.	171	A swarm of slippery micropropellers penetrates the vitreous body of the eye	Review	Microrheology; nanoparticles; composite; diffusion; delivery; surface; bovine;	SCI ADV	2018.11
Wong et al.	169	Guidelines on Diabetic Eye Care: The International Council of Ophthalmology Recommendations for Screening, Follow-up, Referral, and Treatment Based on Resource Settings	Article	Coherence tomographic angiography; major risk factors; panretinal photocoagulation; microvascular density; global prevalence; cataract surgery; older people; low-income; retinopathy; management;	OPHTHALMOLOGY	2018.1
Samara et al.	167	Quantification of Diabetic Macular Ischemia Using Optical Coherence Tomography Angiography and Its Relationship with Visual Acuity	Article	Foveal avascular zone; fluorescein angiography; capillary non-perfusion; normal eyes; retinopathy; density; edema; microcirculation; disruption; perfusion;	OPHTHALMOLOGY	2017.2
Deng et al.	165	Descemet Membrane Endothelial Keratoplasty: Safety and Outcomes A Report by the American Academy of Ophthalmology	Article	Posterior lamellar keratoplasty; prednisolone acetate 1-percent; refractive outcomes; topical steroids; learning curve; graft survival; macular edema; cell density; DMEK; 1st;	OPHTHALMOLOGY	2018.2
Schlegl et al.	162	Fully Automated Detection and Quantification of Macular Fluid in OCT Using Deep Learning	Article	Optical coherence tomography; visual acuity; diabetic retinopathy; anatomic outcomes; subretinal fluid; degeneration; edema; identification; segmentation; ranibizumab;	OPHTHALMOLOGY	2018.4
Scanlon et al.	162	The English National Screening Programme for diabetic retinopathy 2003–2016	Review	Risk assessment; photography; optimization; severity; quality; screening; diabetic retinopathy; blindness	ACTA DIABETOL	2017.6
Wu et al.	158	Myopia Prevention and Outdoor Light Intensity in a School-Based Cluster Randomized Trial	Article	Time spent outdoors; deprivation myopia; ambient illuminance; Singapore children; meta-analysis; risk factors; prevalence; progression; chicks; population;	OPHTHALMOLOGY	2018.8
Fallacara et al.	157	Hyaluronic Acid in the Third Millennium	Review	Molecular weight hyaluronan; sodium hyaluronate; drug delivery; double-blind; *in vitro*; cross-linking; chemical modifications; knee osteoarthritis; tertiary structures; targeted delivery; biological activity; crosslinking; drug delivery; cosmetic; food supplement; functionalization; hyaluronan applications; hyaluronan derivatives; hyaluronan synthases; hyaluronic acid; hyaluronidases; physico-chemical properties	POLYMERS-BASEL	2018.7

### Research hotspots

**Figure 2:**
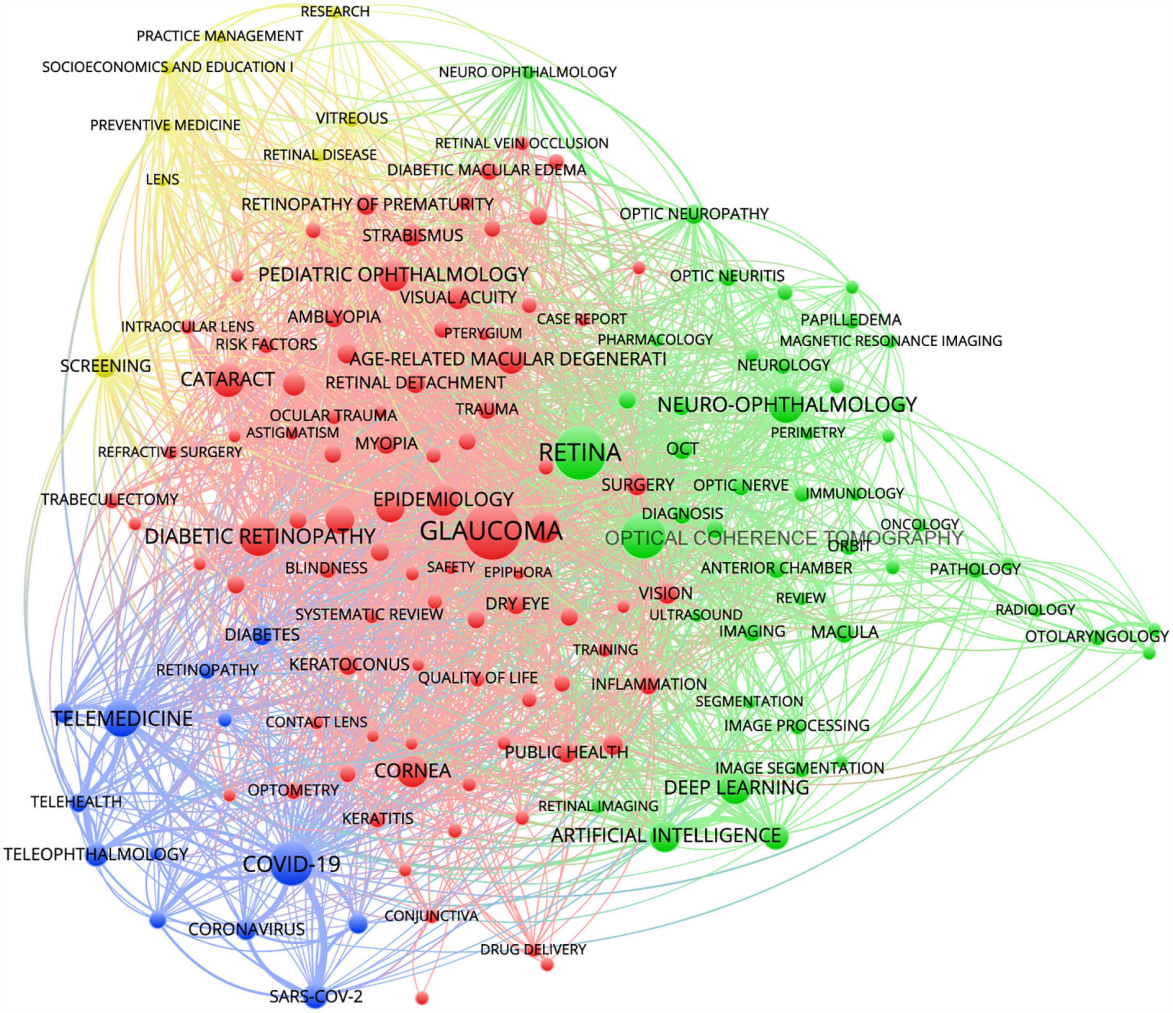
Ophthalmology research hotspots analysis. The keywords formed four clusters, which were differentiated by color in the diagram, with the same color being the same cluster. The keyword size indicated the number of occurrences of the keyword, whereas the thickness and distance of the connecting lines between the keywords indicated the frequency of co-occurrence between the two keywords.

Keyword co-occurrence analysis demonstrated that the three most frequent of all keywords were “glaucoma” (*n* = 395), “retina” (*n* = 321), and “optical coherence tomography” (*n* = 230). In the past 5 years, 157 high-frequency keywords in the field of ophthalmology were identified by setting the minimum frequency of keyword occurrence at 20 times. These keywords formed four clusters: the “glaucoma” cluster (red; 86 items), the “retina” cluster (green; 47 items), the “COVID-19” cluster (blue; 13 items), and the “screening” cluster (yellow; 8 items) (Figure 2). After summarizing the keyword clusters, four research hotspots were identified: epidemiological characteristics and treatment modalities of diseases such as glaucoma and diabetic retinopathy, artificial intelligence and fundus imaging technology, COVID-19-related telemedicine, and screening and prevention of eye diseases.

### Research trends

**Figure 3:**
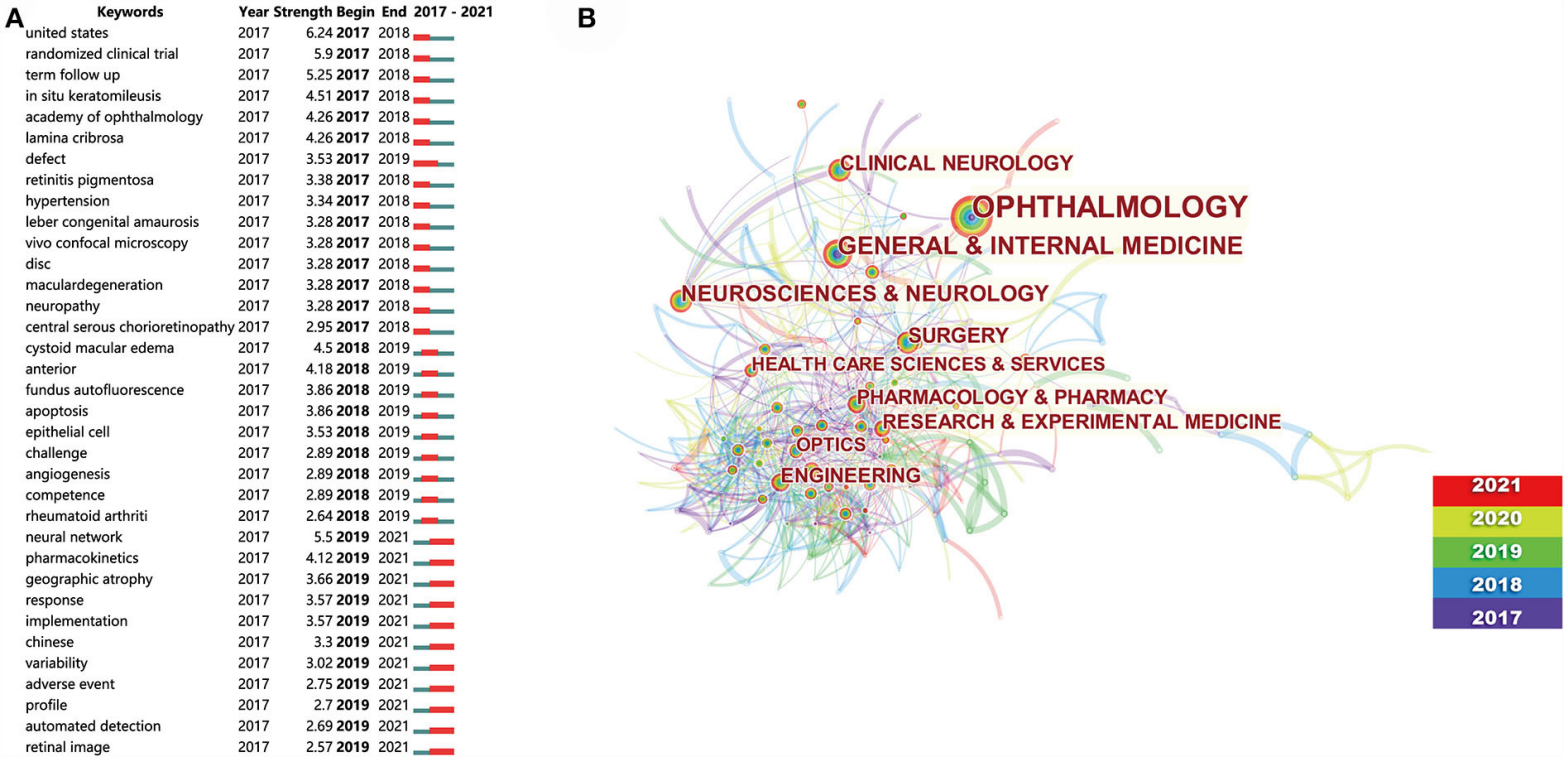
Ophthalmology research trends analysis. **(A)** Keyword burst analysis. The red line indicates the year in which the burst of the corresponding keyword began and ended. **(B)** Subject category analysis. The larger subject categories indicate their greater frequency and importance, and the distance between subject categories indicates how closely they collaborate. The lines between subject categories indicate the collaboration between the subject categories at either end, with the color of the different lines representing the collaboration time in the different subject categories and the thickness representing the degree of collaboration closeness. The color of the temporal rings represents the occurrence of that subject category in different years, the thicker the corresponding temporal rings, the more frequently it occurs, with the time scale at the bottom right.

Keyword burst analysis showed that “neural network,” “pharmacokinetics,” “geographic atrophy,” “implementation,” “variability,” “adverse events,” “automated detection,” and “retinal images” were the hot topics of research in the field of ophthalmology through 2021 and displayed the potential to become the research frontiers to achieve breakthroughs shortly (Figure 3A).

In terms of subject categories, the top three subject categories with the highest volume of ophthalmology-related research publications were medicine general internal (*n* = 1,138, 10.87%), clinical neurology (*n* = 482, 4.604%), and surgery (*n* = 368, 3.515%) (Table 2). The subject categories of ophthalmology research were divided into two types: one was the traditional ophthalmology-related subject categories, such as medicine general internal, clinical neurology, and surgery, and the other one was the non-ophthalmology-related subject categories, such as engineering, computer science, and chemistry. The analysis of subject category collaboration relationships indicated that over time more collaborative relationships had emerged between non-ophthalmology-related subject categories (Figure 3B).

**Table 2:** Subject categories in ophthalmology from 2017 to 2021.

**Category**	**Count**	**Percentage**
Ophthalmology	6,233	59.538
Medicine general internal	1,138	10.870
Clinical neurology	482	4.604
Surgery	368	3.515
Medicine research experimental	288	2.751
Pharmacology pharmacy	275	2.627
Optics	226	2.159
Health care sciences services	223	2.130
Veterinary sciences	203	1.939
Pediatrics	199	1.901
Multidisciplinary sciences	182	1.738
Radiology nuclear medicine medical imaging	151	1.442
Engineering biomedical	144	1.375
Public environmental occupational health	143	1.366
Engineering electrical electronic	119	1.137
Neurosciences	110	1.051
Biochemistry molecular biology	80	0.764
Genetics heredity	80	0.764
Education scientific disciplines	73	0.697
Biochemical research methods	72	0.688
Computer science artificial intelligence	72	0.688
Medical informatics	71	0.678
Rheumatology	69	0.659
Chemistry multidisciplinary	68	0.650
Health policy services	60	0.573

## Discussion

Research in the field of ophthalmology showed a year-on-year increase in the number of articles published in the last 5 years, with the most published country being the United States and the most prolific journal being the Ophthalmology. The top 25 high-impact articles worldwide were cited more than 150 times per article. A total of four research hotspots were identified: epidemiological characteristics and treatment modalities of diseases such as glaucoma and diabetic retinopathy, artificial intelligence and fundus imaging technology, COVID-19-related telemedicine, and screening and prevention of eye diseases. Cross-talk between different non-ophthalmology subject categories was also an important trend in ophthalmology.

The annual publication volume, country distribution, and journal distribution of the ophthalmology research articles revealed a global overview of research output in the field of ophthalmology. The output of ophthalmology research showed an increasing trend in the last 5 years, suggesting that the socioeconomic input and scientific output of the subject area were also developing (20). The individual contributions of some countries to ophthalmology research were previously reported, but there were limitations on the overall evaluation of all countries' contributions to ophthalmology research and of country collaboration (13–17). This study showed that the predominant countries in ophthalmology research included the United States, the United Kingdom, and India, and countries such as Germany, China, and Australia also played an important role in the contribution. Several stable collaborative networks have been formed between countries, which can facilitate cross-border research data sharing and the globalization of scientific research. The top five most published journals showed that ophthalmology research was mainly focused on clinical ophthalmology (Ophthalmology, BMJ Case Reports), basic ophthalmology research (Investigative Ophthalmology and Visual Science) and neuro-ophthalmology (Journal of Neuro-Ophthalmology, Ophthalmology Retina).

The high-impact articles in ophthalmology indicated that researchers in the field of ophthalmology were primarily concerned with ophthalmological health or disease states, as well as ophthalmological technologies and applications. In terms of health or disease conditions, age-related macular degeneration (21–24), glaucomatous optic neuropathy (25, 26), corneal blindness (27), and other blinding eye diseases occupied important research positions. Research directions such as screening for diabetic retinopathy (28, 29), preventing myopia (30), optimizing visual outcomes, and controlling complications after IOL implantation following cataract surgery were dedicated to the active identification, management, and control of disease risk factors, making the eye disease controllable and manageable (31, 32). In addition, researchers were also concerned with the management of Behcet's syndrome (33) and COVID-19 infection prevention in ophthalmology (34). In ophthalmology-related technologies, the frontiers were artificial intelligence algorithms (23, 25, 26, 35–38), new pathways for drug delivery (39, 40), and new materials for therapy (41). In ophthalmology-related applications, the pioneering applications were optical coherence tomography (23, 24, 35, 42–44), stem cell therapy, and tissue repair (45).

After clustering the high-frequency keywords in the past 5 years, four research hotspots in the field of ophthalmology were obtained. First, the epidemiological characteristics and treatment modalities of diseases such as glaucoma and diabetic retinopathy were the hot topics of ophthalmology research. The emergence of these hot topics was consistent with the increasing prevalence of systemic chronic diseases such as diabetes in the last 5 years, and several studies have revealed associations and common biomarkers of ophthalmology and systemic diseases (46–49). More future work needs to further focus on the diagnosis and optimal treatment strategies for blinding diseases associated with systemic conditions (50). Moreover, deep learning algorithms that could rapidly and non-invasively identify pathological features of eye diseases joined ophthalmology research (23). Deep learning algorithms could classify age-related cataract types based on slit-lamp photographs, and fully automated AI-based screening systems had been approved for the use in diabetic retinopathy (37, 51). Furthermore, the emergence of the COVID-19 pandemic brought about an increase in the length of patient visits due to disease control and health-related problems associated with COVID-19 infections, which had a dramatic impact on ophthalmology health care. On the one hand, the close contacts physicians need when attending to patients could increase the risk of cross-infection between patients or between health care workers and patients, resulting in infection control to be optimized in ophthalmology practice. On the other hand, the need for timely intervention for patients was driving the development of telemedicine during the pandemic (34, 52). Finally, the development of diagnostic technology has driven ophthalmology research toward early screening and disease prevention.

The keywords that were still bursting until 2021 were research trends. The keywords “neural networks,” “pharmacokinetics,” “automated detection,” and “retinal images” in this part of the keyword list were consistent with the hot research directions obtained by keyword clustering. Other keywords that had burst to 2021 could be newly emerging keywords that had not yet had time to be highly cited, were hotspots for research in ophthalmology, and were likely to continue to be of interest for some times to come. Concerning the disciplinary analysis, the analysis of this study revealed that there was extensive cross-collaboration in various basic areas of non-ophthalmology-related research. Knowledge from non-ophthalmology fields is likely to be more involved in ophthalmology research.

Strengths of the study include a global view of research forces in ophthalmology from a wide range of the literature. Additional study strengths include the revealing of highly cited documents in ophthalmology that provide useful information for researchers. Outcome measures addressed the global research force contributions, research hotspots, and research trends of ophthalmology research, providing an in-depth study of the field of ophthalmology.

Only data from the Web of Science Core Collection database were included in this study, but the Web of Science Core Collection database, as a citation database, already contained comprehensive data on the articles and corresponding citations, which was sufficient for capturing the overall development of the scientific field. In addition, the results of the analysis by the visualization software may include some repetitive and meaningless information. We tried to identify some of the hot topics that were influencing ophthalmology research, so the raw data had been further filtered to remove irrelevant or meaningless words.

In conclusion, this study provided a comprehensive analysis of ophthalmology-related research based on the Web of Science Core Collection database. The hotspots in ophthalmology research were epidemiology, prevention, screening, and treatment of ocular diseases, as well as artificial intelligence and fundus imaging technology and telemedicine. Research trends in ophthalmology research were artificial intelligence, drug development, and fundus diseases. There was an extensive cross-talk of ophthalmology-related research in various basic areas. Knowledge from non-ophthalmology fields is likely to be more involved in ophthalmology research.

## Data availability statement

Publicly available datasets were analyzed in this study. This data can be found here: https://www.webofscience.com/wos/alldb/basic-search.

## Author contributions

ZL and GJ designed the study and provided a critical review for the manuscript. YT and WZ wrote the manuscript. YT, WZ, YZ, BZ, YY, and WL collected and analyzed the data. All authors contributed to the article and approved the submitted version.

## Funding

This study was supported by the National Natural Science Foundation of China (81873675), the Guangdong Basic and Applied Basic Research Foundation (2022A1515011181), the Teaching Reform Research Program of Sun Yat-sen University (JX3030604024), and the Youth Project of State Key Laboratory of Ophthalmology (2021QN02).

## Conflict of interest

The authors declare that the research was conducted in the absence of any commercial or financial relationships that could be construed as a potential conflict of interest.

## Publisher's note

All claims expressed in this article are solely those of the authors and do not necessarily represent those of their affiliated organizations, or those of the publisher, the editors and the reviewers. Any product that may be evaluated in this article, or claim that may be made by its manufacturer, is not guaranteed or endorsed by the publisher.
